# Effects of ammonia exposure on the expression of *IL-1β*, *CRH*, and *lep-a1* genes in common carp (*Cyprinus carpio*)

**DOI:** 10.1186/s12917-025-04749-1

**Published:** 2025-05-28

**Authors:** Mahmoud Nasr, Mohammed Youssef, Abdullah A. A. Alghamdi, Ali H. Alghamdi, Mohsen A. Khormi, Ali M. Aborasain, Walaa F. A. Emeish, Ahmad A. Elkamel

**Affiliations:** 1 Department of Fish Diseases, Faculty of Veterinary Medicine, South Valley University, Qena, Egypt; 2 Department of Animal Physiology, Faculty of Veterinary Medicine, South Valley University, Qena, Egypt; 3 Department of Biology, Faculty of Science, Al-Baha University, Al-Baha, Saudi Arabia; 4 Department of Biology, College of Science, Jazan University, P.O. Box. 114, Jazan, 45142 Saudi Arabia; 5 Department of Aquatic Animal Medicine and Management, Faculty of Veterinary Medicine, Assiut University, Assiut, Egypt

**Keywords:** *Cyprinus carpio*, Un-ionized ammonia, RT-qPCR, Pro-inflammatory cytokines, Stress response, Leptin

## Abstract

**Background:**

Common carp is one of the most economically important freshwater fish species globally. Ammonia exposure, a frequent challenge in aquaculture, can lead to significant economic losses. This study investigated the impact of un-ionized ammonia (UIA) exposure on the expression profiles of three key genes in common carp fry: interleukin-1 beta (*IL-1β*), corticotropin-releasing hormone (*CRH*), and leptin a1 (*Lep-a1*). These genes are crucial indicators of immune response, stress regulation, and appetite control, respectively. Fish were exposed to 0.7 mg/L of UIA, and gene expression was analysed in liver and gill tissues at five time points (12 h, 2-, 4-, 7-, and 14-days of exposure) using quantitative real-time PCR (RT-qPCR).

**Results:**

Results demonstrated that expression levels of all three genes were significantly affected by exposure time and tissue type. *IL-1β*,* CRH*, and *Lep-a1* were upregulated in both liver and gill tissues, with the liver consistently showing higher expression levels. Notably, significant positive correlations were observed between each pair of the three genes studied, suggesting a coordinated physiological response to ammonia stress. The liver emerged as a key organ in orchestrating the long-term adaptive response, while the gills exhibited a more acute, transient reaction.

**Conclusions:**

This study provides valuable insights into the molecular mechanisms underlying the physiological response of common carp to ammonia toxicity. The findings highlight the complex interplay between immune, stress, and metabolic pathways in coping with ammonia exposure. A deep understanding of these mechanisms could lead to improved management strategies in aquaculture and the development of potential biomarkers for assessing stress responses in fish populations.

## Background

Common carp (*Cyprinus carpio*) holds a significant position in global aquaculture, ranking fourth in production as of 2018 with 7.7% of the total yield, following Nile tilapia (*Oreochromis niloticus*), silver carp (*Hypophthalmichthys molitrix*), and grass carp (*Ctenopharyngodon idella*) [1]. Beyond its importance in aquaculture, common carp serves as a valuable bio-indicator for toxicological studies due to its high sensitivity to toxic substances and rapid response to changes in the aquatic environment [2].

Nitrogenous compounds extensively affect the health of fish [3]. Ammonia toxicity represents a major environmental challenge for fish populations worldwide, often leading to cellular swelling – particularly of brain astrocytes and hepatocytes, acidosis, production of reactive oxygen species (ROS), and oxidative damage [4, 5]. These effects can lead to respiratory disorders such as flaring opercula, gasping, asphyxia, and finally fish mortality [6]. The accumulation of ammonia in aquatic environments, particularly in aquaculture settings, is primarily attributed to fish nitrogen metabolism, decomposition of organic matter or uneaten feed, and high stocking densities. Ammonia toxicity of fish can be rapid or slow, dependent on its elevated environmental ammonia levels and exposure duration as well as impairing conditions such as low ambient dissolved oxygen, increased pH or salinity levels, and warm temperature [7].

In aquatic systems, total ammonia exists in an equilibrium between two forms: ionized ammonium (NH_4_^+^) and un-ionized ammonia (NH_3_) [8], with the balance determined by pH and temperature [9]. The equilibrium can be represented as: NH_3_ + H_3_O^+^ ⇔ NH_4_^+^ + H_2_O. NH_3_ is considered highly toxic due to its uncharged and lipid-soluble nature, allowing it to permeate epithelial membranes such as those in fish gills readily [10]. In contrast, NH_4_^+^ is relatively non-toxic to fish due to its larger, hydrated, and charged form, which impedes its passage through the hydrophobic micropores of gill membranes [10]. The ratio of NH_3_ to NH_4_^+^ in water depends on pH, temperature, with NH_3_ becoming more abundant at higher temperature and/or pH [11].

Ammonia exposure has a significant impact on fish immune responses, particularly through modulation of cytokine expression [9]. Environmental stressors or infections can stimulate or inhibit the expression of innate immune-related cytokines. Interleukin-1β (IL-1β) is a crucial cytokine that triggers cellular inflammatory immune responses and works in concert with tumor necrosis factor-alpha (TNF-α) to regulate immune responses, including proliferation, differentiation, chemotaxis, and activation of both nonspecific and specific immune responses [12]. Previous studies have investigated the impact of ammonia exposure on *IL-1β* gene expression in the kidney of crucian carp (*Carassius auratus*) [13] and the liver of turbot (*Scophthalmus maximus*) [14].

Exposure to ammonia in various fish species has been associated with increased plasma cortisol levels, in teleosts, the neurosecretory pre-optic hypothalamic area responds to stress by releasing corticotropin-releasing hormone (CRH), which is the primary regulator of the HPI axis. CRH stimulates the anterior pituitary gland to release adrenocorticotropic hormone (ACTH) which in turn prompts the interrenal cells in the head kidney to release cortisol [15].

High ammonia levels can lead to various adverse physiological effects, including reduced growth and food intake in fish [16]. Leptin (Lep-a1), an anti-obesity hormone with anorexigenic properties, is produced by the obesity gene (*ob* or *Lep*) [17]. While leptin is primarily secreted by adipose tissue in mammals [18], the liver is the main source of leptin in fish [19]. Although ammonia has been shown to have an anorexigenic effect [20], the molecular mechanisms by which ammonia poisoning influences feed intake in fish have not been thoroughly elucidated.

Despite the importance of common carp in aquaculture and its use as a bio-indicator, there was a paucity of information regarding the effects of ammonia exposure on the expression of *IL-1β*,* CRH*, and *Lep-a1* genes in this species. Therefore, this study aimed to investigate the expression profiles of immune (*IL-1β*), stress (*CRH*), and appetite (*Lep-a1*)-related genes in common carp following a 14-day exposure to ammonia. The findings of this research were intended to contribute to a better understanding of the molecular responses of common carp to ammonia stress and to potentially inform strategies for improving aquaculture practices and environmental monitoring.

## Results

### Clinical examination

**Fig. 1:**
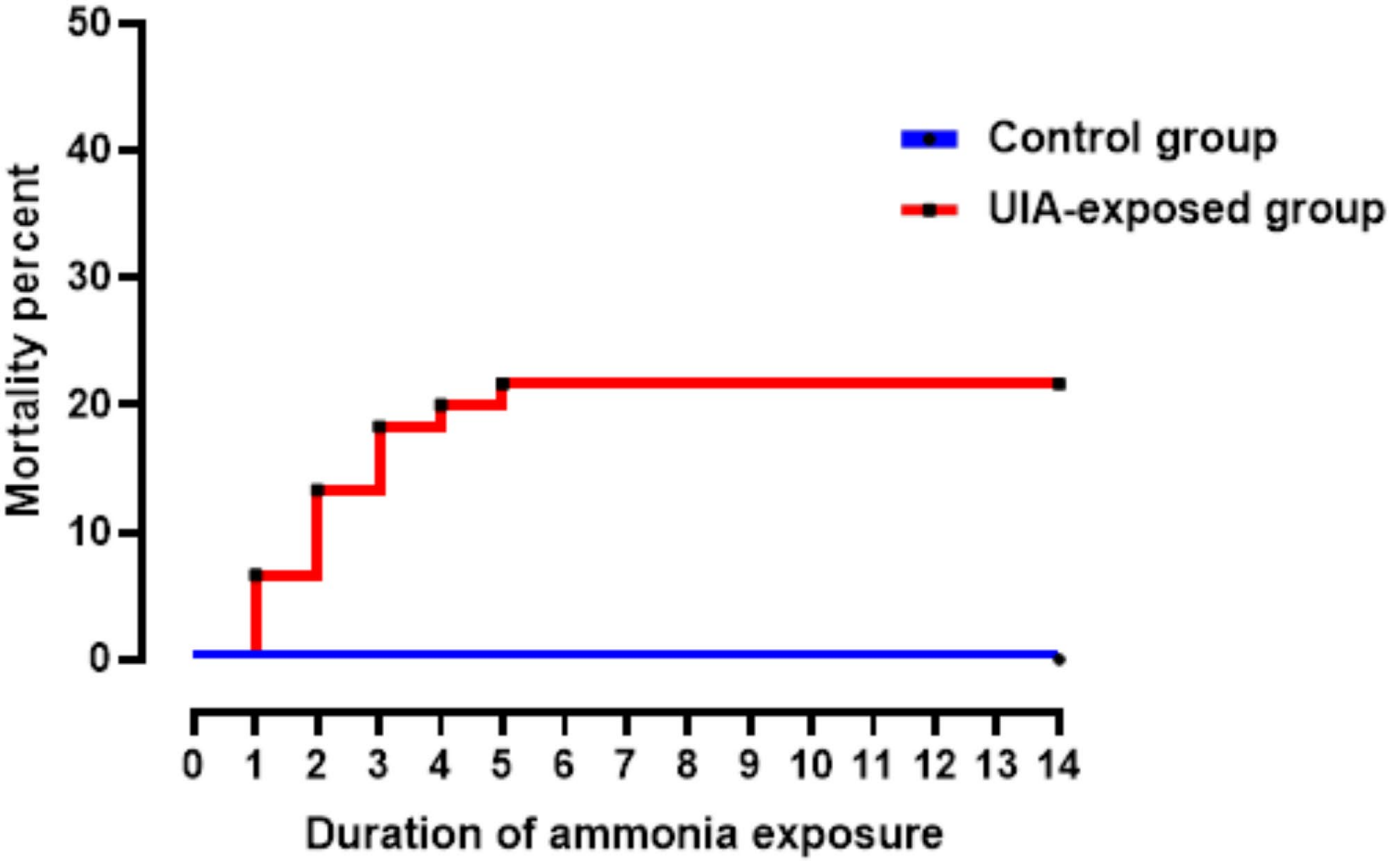
Cumulative mortality percentage of common carp over the 14-day experimental period in the control group and the group exposed to 0.7 un-ionized ammonia (UIA)

Throughout the two-week experimental period, fish in the control group exhibited no lesions or mortality. In contrast, the UIA-exposed group demonstrated a cumulative mortality rate of 21.67% by the end of the experiment (Fig. 1). The mortality curve shows a rapid increase in deaths during the first 5 days of exposure, followed by a plateau for the remainder of the study period.

Un-ionized ammonia-exposed fish displayed a range of behavioural and physical changes. Initially, they became lethargic and dull, with a noticeable decrease in feeding appetite, evidenced by the accumulation of uneaten feed in the tanks. However, a partial recovery in appetite was observed towards the end of the exposure period. Clinical examination of UIA-exposed fish revealed several distinct pathological signs, including erythematous streaks on the body and fins, progressive fin erosion corresponding to the duration of exposure, and skin damage with scale loss. Post-mortem examination of deceased fish uncovered additional pathological changes. This included gill congestion, characterized by red or purple coloration which later turned pale, damage to gill filaments and overall gill swelling, and excessive mucous production in the gills. The fish also exhibited significant loss of body weight, darkening of the skin, signs of hypoxia, and a nearly empty gastrointestinal tract.

### Expression pattern of the genes under study

**Fig. 2:**
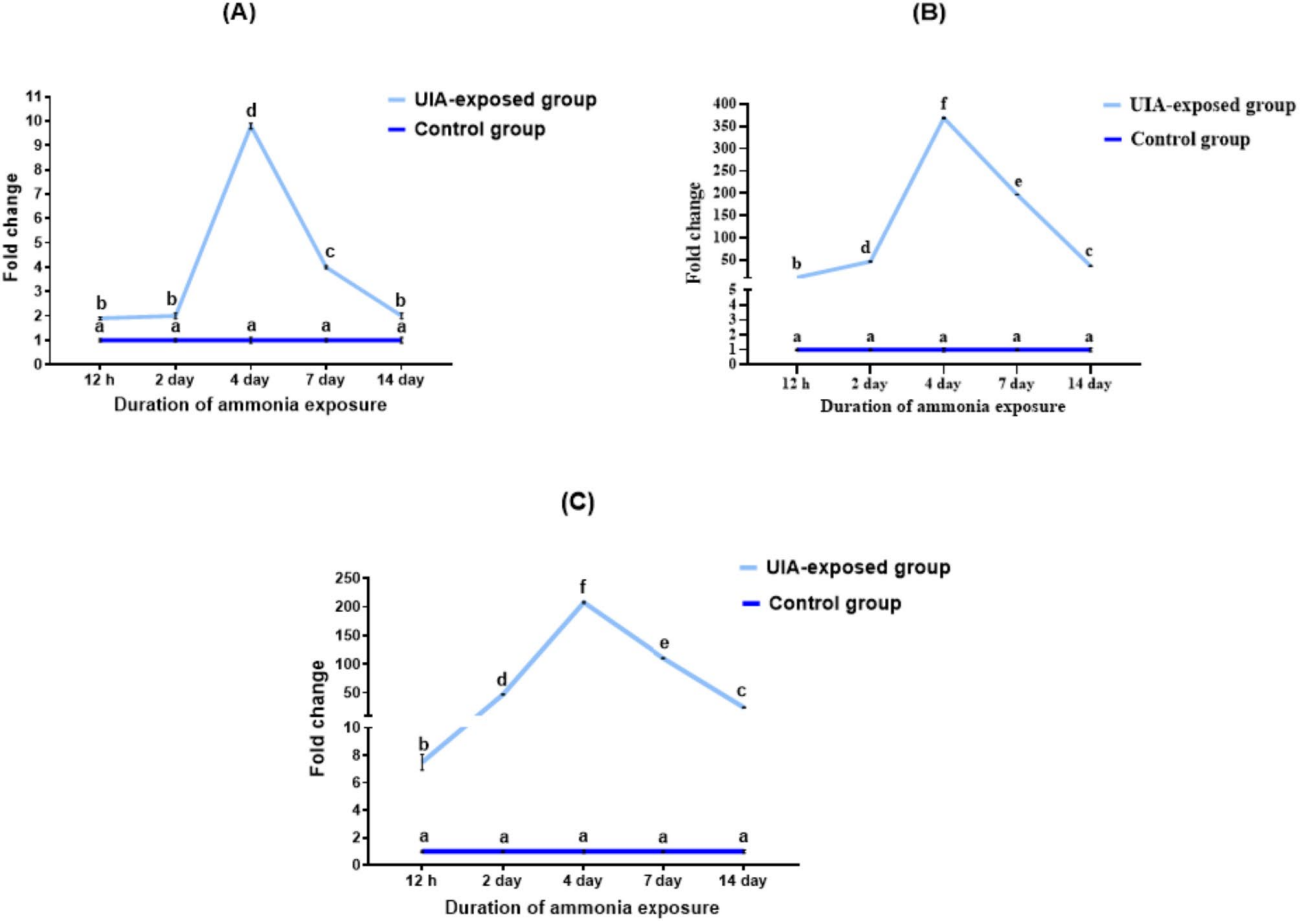
Line plots showing the expression profiles of *IL-1β* (**A**), *CRH* (**B**), and *Lep-a1* (**C**) genes in the liver of common carp exposed to 0.7 mg/L of un-ionized ammonia over 12 h, and 2, 4, 7, and 14 days. Expression levels were normalized to *EF1α* and *β-actin* genes. Data are presented as mean ± standard error of the mean. Statistically significant differences in expression levels are indicated by different lowercase letters (two-way ANOVA, *P* < 0.05)

The temporal expression profiles of *IL-1β*,* CRH*, and *Lep-a1* genes in the liver of common carp exposed to UIA demonstrated a coordinated response pattern (Fig. 2A, B, and C). All three genes showed gradual upregulation starting at 12 h of exposure, peaking at day 4, followed by a decline while remaining significantly elevated compared to controls throughout the 14-day experiment. *IL-1β* (Fig. 2A) reached a maximum 9.8-fold change at day 4. *CRH* (Fig. 2B) exhibited the most dramatic increase with a 368.4-fold change at day 4. *Lep-a1* (Fig. 2C) peaked at day 4 recording 207.9-fold change.

**Fig. 3:**
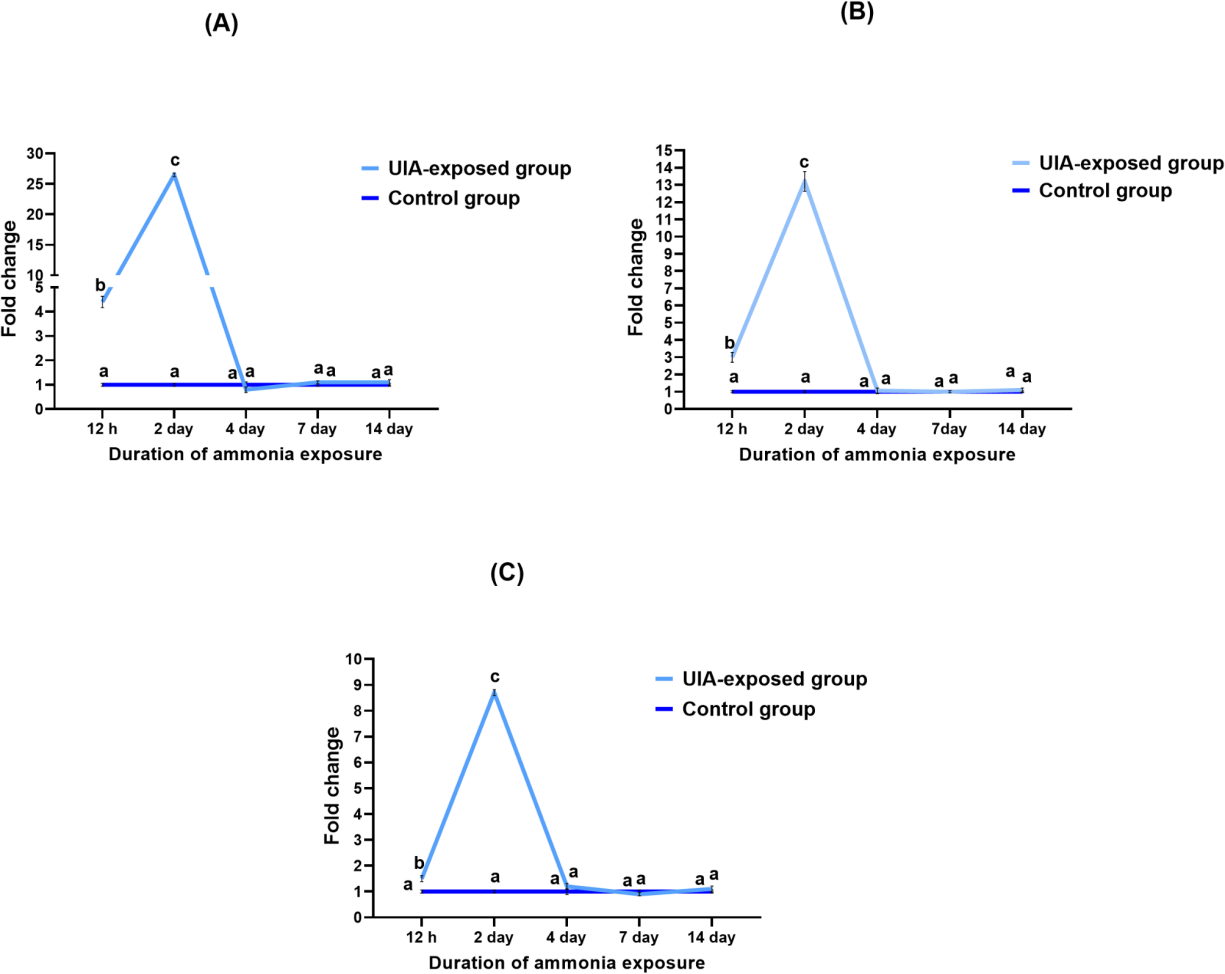
Line plots showing the expression profiles of *IL-1β* (**A**), *CRH* (**B**), and *Lep-a1* (**C**) genes in the gills of common carp exposed to 0.7 mg/L of un-ionized ammonia over 12 h, and 2, 4, 7, and 14 days. Expression levels were normalized to *EF1α* and *β-actin* genes. Data are presented as mean ± standard error of the mean. Statistically significant differences in expression levels are indicated by different lowercase letters (two-way ANOVA, *P* < 0.05)

In the gills, *IL-1β*,* CRH*, and *Lep-a1* gene expressions exhibited a coordinated response to UIA exposure (Fig. 3A, B, and C). All three genes showed significant upregulation within 12 h of exposure, reaching peak expression levels at day 2 before returning to baseline levels. *IL-1β* (Fig. 3A) demonstrated a 26-fold increase at day 2, indicating a robust inflammatory response in the gills. *CRH* (Fig. 3B) showed the highest relative upregulation with a 13.2-fold change, suggesting acute activation of the stress response pathway. *Lep-a1* (Fig. 3C) exhibited an 8.7-fold increase, potentially reflecting alterations in local metabolic regulation. Notably, expression levels for all three genes rapidly declined after day 2, returning to control levels by day 4 and remaining at baseline through day 14.

**Fig. 4:**
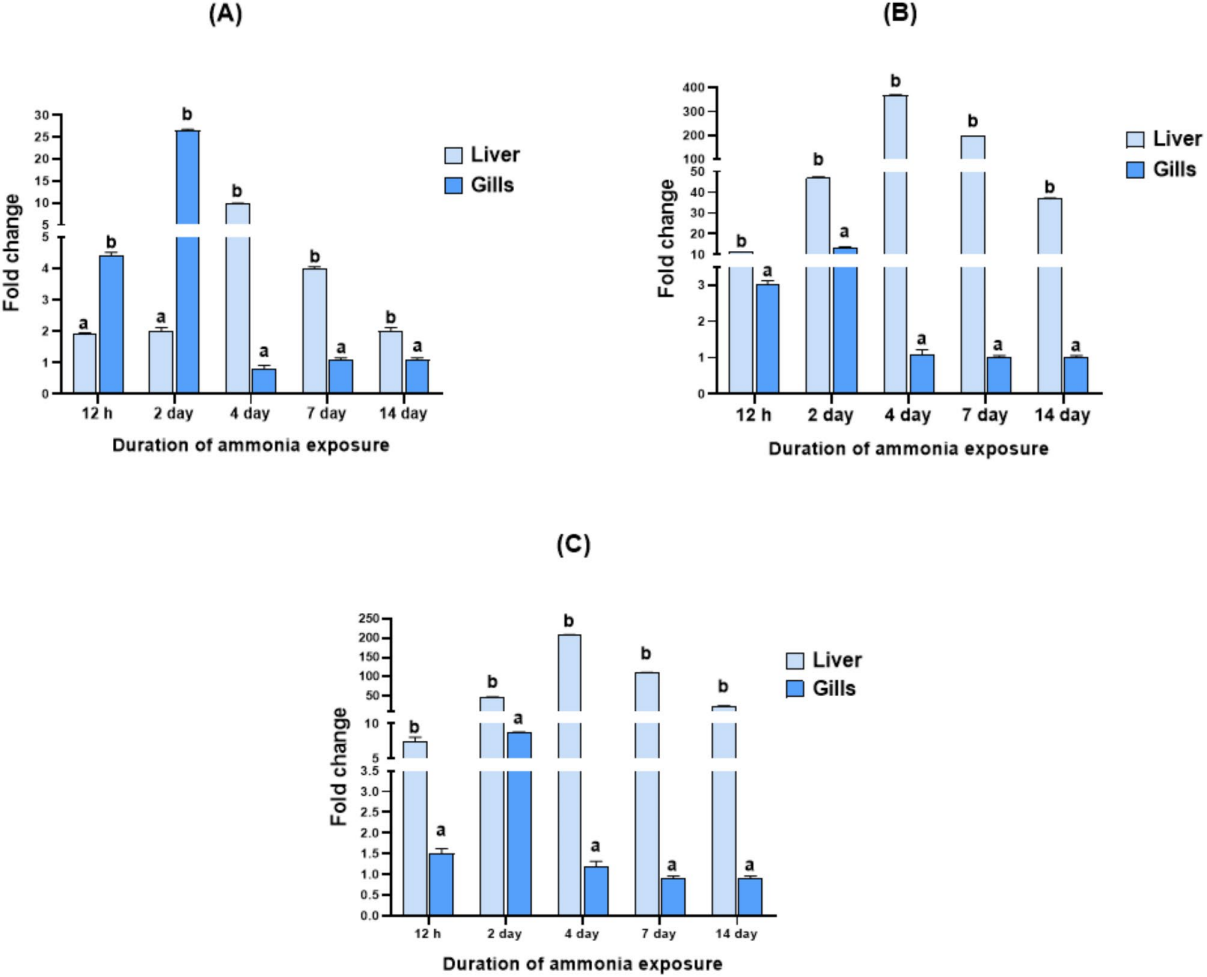
Expression profiles of *IL-1β* (**A**), *CRH* (**B**), and *Lep-a1* (**C**) genes relative to *EF1α* and *β-actin* as housekeeping genes in the liver and gills of common carp exposed to 0.7 mg/L of un-ionized ammonia for 12 h, and 2, 4, 7, and 14 days. Data are presented as mean ± standard error of the mean. Statistically significant differences in expression levels are indicated by different lowercase letters (two-way ANOVA, *P* < 0.05) within a given time point

The expression profiles of *IL-1β*,* CRH*, and *Lep-a1* genes revealed distinct patterns. Specifically, the expression of *IL-1β* was significantly elevated in the gills compared to the liver after 12 h and 2 days of UIA exposure. This differential expression trend then reversed at the 4-, 7-, and 14-day time points, with *IL-1β* exhibiting higher expression in the liver than in the gills. In contrast, both *CRH* and *Lep-a1* consistently displayed significantly higher expression in the liver than in the gills across all time points of UIA exposure (Fig. 4).

**Fig. 5:**
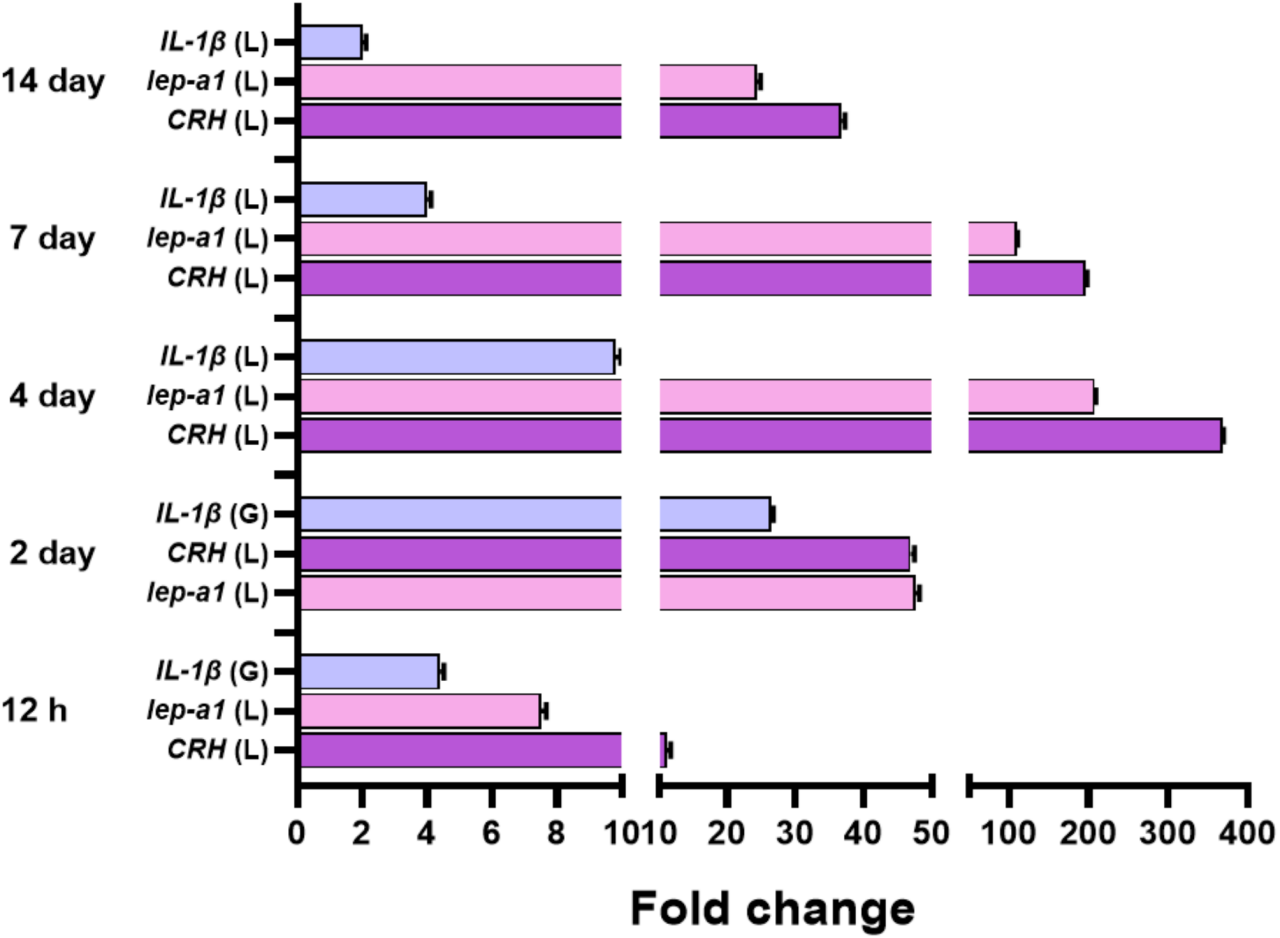
Maximum fold change of *IL-1β*, *CRH*, and *Lep-a1* genes in the liver and gills of common carp exposed to 0.7 mg/L of un-ionized ammonia over 12 h, and 2, 4, 7, and 14 days. Data are presented as mean ± standard error of the mean. “L” and “G” refer to the examined tissues, liver and gills, respectively

**Fig. 6:**
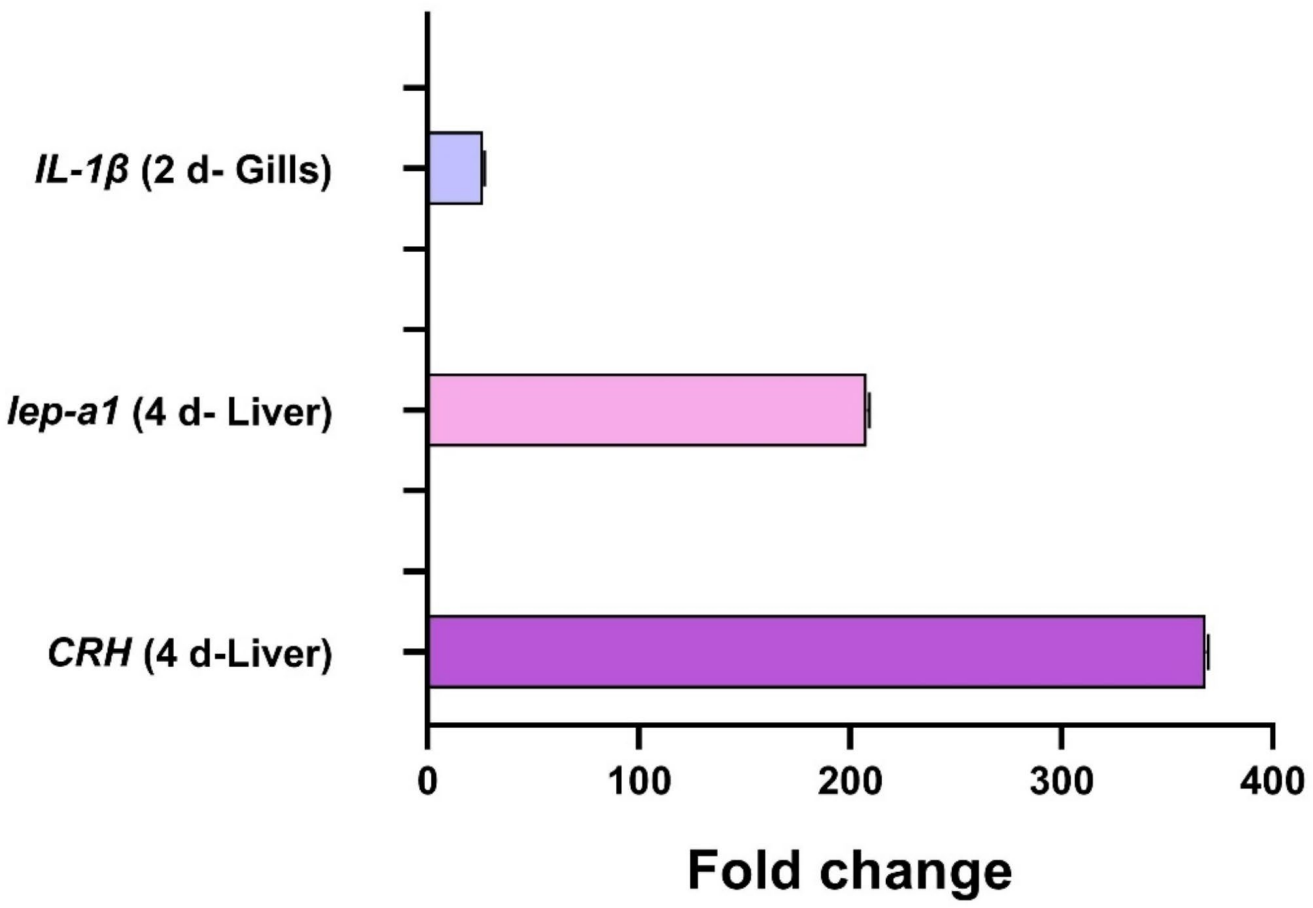
Maximum fold change of *IL-1β*, *CRH*, and *Lep-a1* genes in the liver and gills of common carp exposed to 0.7 mg/L of un-ionized ammonia. Data are presented as mean ± standard error of the mean

The comprehensive analysis of the expression patterns of* IL-1β*,* CRH*, and *Lep-a1* genes across the liver and gills throughout the UIA exposure experiment provides valuable insights into the tissue-specific stress responses (Fig. 5). Notably, the liver exhibited the maximum expression levels for all three genes at the majority of the examined-time points. The gills exhibited the maximum expression of the pro-inflammatory cytokine *IL-1β* at the 2-day time point. In contrast, the liver showed the greatest upregulation of the hypothalamic-pituitary-interrenal axis regulator *CRH* and the metabolic hormone *Lep-a1* at the 4-day time point (Fig. 6).

**Fig. 7:**
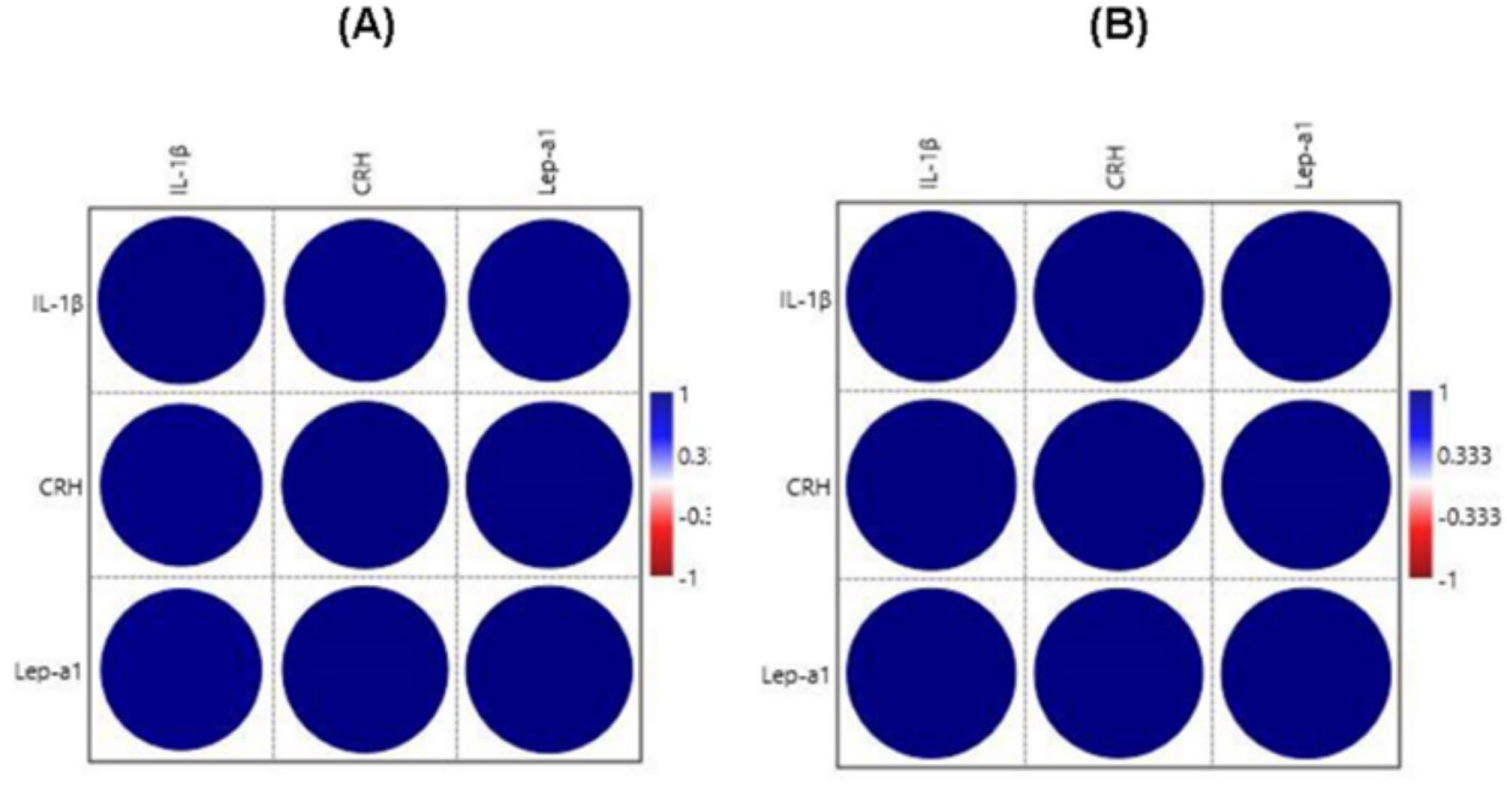
Correlation analysis of *IL-1β*, *CRH*, and *Lep-a1* gene expression in the liver (**A**) and gills (**B**) of common carp exposed to 0.7 mg/L of un-ionized ammonia at 12 h, and 2, 4, 7, and 14 days. Positive correlations are represented by blue circle plots, while negative correlations are indicated by red circles. The strength of the correlation is reflected by the size and color intensity of the circles. Crossed circle plots denote no significant difference (*P* > 0.05)

The correlation analysis revealed a significant positive relationship of the expression between each two of the genes studied in both the liver and gills of exposed common carp (Fig. 7). In the liver, the strong positive correlations between all three genes, as indicated by the large, dark blue circular plots (Fig. 7A). A similar pattern of positive correlations was observed in the gills (Fig. 7B), pointing to a synchronized activation of these key stress-related genes.

## Discussion

Industrial and biological waste decomposition, and agricultural run-off have toxic effects on aquatic organisms [21] with ammonia is the second highest synthesized chemical on ground [22] and is one of the most detrimental water quality parameters for fish. Fish degrade protein and synthesize ammonia in the mitochondrial matrix and cytosol of cells and in the gut, where microbes participate in their digestion [23].

While the negative effects of ammonia toxicity on fish appetite, growth performance, immune status, and stress resistance are well-documented, the molecular-level impacts remain largely unexplored. To elucidate the toxicological effects of ammonia exposure in common carp, this study focused on three genes: *IL-1β*,* CRH*, and *Lep-a1*.

The present results emphasized that *IL-1β* upregulation in the liver and gills in response to ammonia exposure implies induction of inflammation. Thus, *IL-1β* stimulates a pro-inflammatory response to regulate immunity and manage ammonia exposure in common carp. These findings align with a previous study observed significant upregulation of *IL-1β* gene expression in turbot liver after 96 h of exposure to 20 or 40 mg/L TAN [14]. Similarly, in Asian clam (*Corbicula fluminea*), gill IL-1β protein content increased between 24 and 48 h following exposure to 25.0 mg/L of aerial ammonia [4]. In crucian carp, exposure to 10 mg/L of ammonia led to upregulation of *IL-1β* in the kidney after 15 days, but downregulation after 30 days [13]. The observed upregulation of *IL-1β* expression in ammonia-exposed fish may be attributed to the stimulation of neutrophil alkaline phosphatase 3-independent inflammatory factors. These factors trigger caspases to promote the maturation of pro-IL-1β, inducing an inflammatory response [24] and activating additional inflammatory molecules to initiate further inflammatory processes, as previously reported [25].

The upregulation of *IL-1β* gene expression observed in the gills of common carp in the current experiment may be due to their direct and continuous exposure to ammonia throughout the study suggesting an acute immune and stress response in this primary site of UIA exposure. The local and direct response of gills to pathogens and chemical and physical stress is crucial for maintaining internal homeostasis, as previously reported [26]. Exposure to high ambient ammonia levels can result in ammonia uptake from the environment, elevating internal ammonia levels and/or inhibiting branchial ammonia excretion [27].

In this study, ammonia exposure stimulated *CRH* expression in both liver and gills, indicating a clear activation of the hypothalamic-pituitary-interrenal (HPI) axis in common carp and highlighting the relationship between the functional status of different organs and the brain. Cytokines can affect the activity of the HPI axis, with *IL-1β*, *TNF-α*, and to a lesser extent, interleukin-6 (IL-6) considered the main factors in this communication between the HPI axis and the immune system [28]. This relationship can explain the enhanced co-expression of both *IL-1β* and *CRH* in a time-dependent manner in the liver and gills observed in the current experiment. These findings suggest that *IL-1β* enhances the adrenocorticotropic hormone (ACTH) cells’ response to *CRH*, as previously suggested [29], and supports the potential crosslinks between inflammation and stress occurrence. Apart from gene expression, a previous study showed that, after being exposed to 40 mg/L of TAN for 24 and 48 h, the plasma of turbot had a significant rise in *CRH*, but after 96 h, the value had returned to the control [30]. While in the HYP of rainbow trout, ammonia exposure (1000 µmol/L) was associated with an upregulation in *CRH* gene levels in the brain at 96 h [31].

Leptin, an unglycosylated 16 KDa multifunctional hormone belonging to the cytokine superfamily, is responsible for regulating stress responses, reproduction, growth, body weight, and food consumption [32]. It has been described in several fish species, including common carp, and is primarily expressed in the liver [33]. In this study, the reduced feeding behaviour in the exposed group, which is a common stress response in fish, aligning with the stimulation of *Lep-a1* gene expression as an appetite suppressant in response to ammonia exposure. Ammonia stimulated *Lep-a1* gene expression primarily in the liver, followed by the gills [34], potentially through a negative feedback loop to the hypothalamic appetite center [35]. This response may protect fish from significant postprandial elevations in plasma ammonia levels and minimize ammonia toxicity [36].

This study demonstrated that *CRH* and *Lep-a1* exhibited similar expression patterns during ammonia exposure. This may be because both *CRH*, as a regulator of the HPI axis, and *Lep-a1* are potent appetite suppressors in fish during stress responses [37]. Additionally, cortisol stimulates the release of leptin [38]. Therefore, the changes in food intake during stress observed in this study may be linked to the activation of the leptin system [39].

In the current study, it is noteworthy that all clinical signs, post-mortem lesions, and mortalities were predominantly observed within the first week of UIA exposure and the gradual recovery of appetite with the cessation of mortalities during the second week of the experiment were associated with the return of *IL-1β*,* CRH*, and *Lep-a1* gene expressions to control levels, especially in the gills. However, the expression levels of these genes remained significantly elevated in the liver after 14 days of exposure. This heightened expression in the liver underscores the central role of this organ in coordinating the integrated physiological stress response to UIA toxicity, likely through immune, endocrine signalling and metabolic adjustments. This pattern suggests an acute, transient response in the gills to UIA exposure, contrasting with the more sustained response observed in the liver, suggesting a potential adaptive response or increased tolerance to the UIA exposure over time through converting ammonia to a less toxic compound, such as urea, through the urea cycle in the liver [40]. Alternatively, the fish may have started to form glutamine in the brain by reacting with glutamate to detoxify ammonia [41].

The findings of the current study strongly suggest a level of coordination or crosstalk between the three genes studied, as indicated by the significant positive correlation observed among them supporting a coordinated upregulation of these stress-responsive genes. The consistent positive correlations between *IL-1β*,* CRH*, and *Lep-a1* in both the liver and gills underscores the intricate interconnectedness of the physiological pathways governing the fish’s adaptive response to ammonia toxicity.

## Methods

### Fish

A total of 315 common carp fry, weighing 2 ± 0.5 g, were purchased from a private fish farm in Dandara, Qena Governorate, Egypt, and transported to the aquatic laboratory of the Fish Diseases Department, Faculty of Veterinary Medicine, South Valley University. Random fish samples were examined before experimentation to confirm their health and exclude abnormalities. The fish were acclimated for 14 days in 500-L holding tanks filled with dechlorinated tap water and continuously aerated. Water quality was maintained at total ammonia (0.11 ± 0.03 mg/L), pH (7.17 ± 0.24), temperature (25 ± 0.5 °C), and dissolved oxygen (6.1 ± 0.52 mg/L).

### Pilot toxicity experiment

A pilot toxicity experiment was conducted to identify a toxic concentration of un-ionized ammonia (UIA) that would elicit clinical responses in common carp fry without causing more than 40% mortality. Five UIA concentrations (0.5, 0.7, 0.9, 1.1, and 1.3 mg/L) were prepared using reagent-grade ammonium chloride (Merck, Germany) and dechlorinated water. NH_3_ levels were calculated based on total ammonia nitrogen (TAN), pH, and temperature using Thurston’s method [42]. Fish were exposed to these concentrations in 70-L tanks (*n* = 9/tank) for two weeks, with clinical signs and mortality recorded daily. Each test was performed in triplicate. Results showed that 0.7 mg/L UIA was optimal for subsequent experiments, producing clinical effects while limiting mortality.

### Experimental groups and ammonia challenge

Fish were divided into three groups (*n* = 20/group) and housed in 70-L tanks. Two groups were exposed to 0.7 mg/L UIA for 14 days, while the third group, serving as a control, was maintained under the same conditions without UIA exposure. This experiment was performed in triplicate. Mortality rates and clinical signs were recorded from the first exposed group, while fish samples were collected from the second exposed group and the control group at designated intervals.

Un-ionized ammonia concentrations in tanks were monitored every 6 h, with one-quarter of the tank water replaced daily to maintain levels. Tanks were continuously aerated and cleaned daily. Fish were fed a commercial diet containing 30% protein and 6% lipid (Grand Aqua, Egypt) at 3% body weight divided into two daily meals.

### Sample collection

**Fig. 8:**
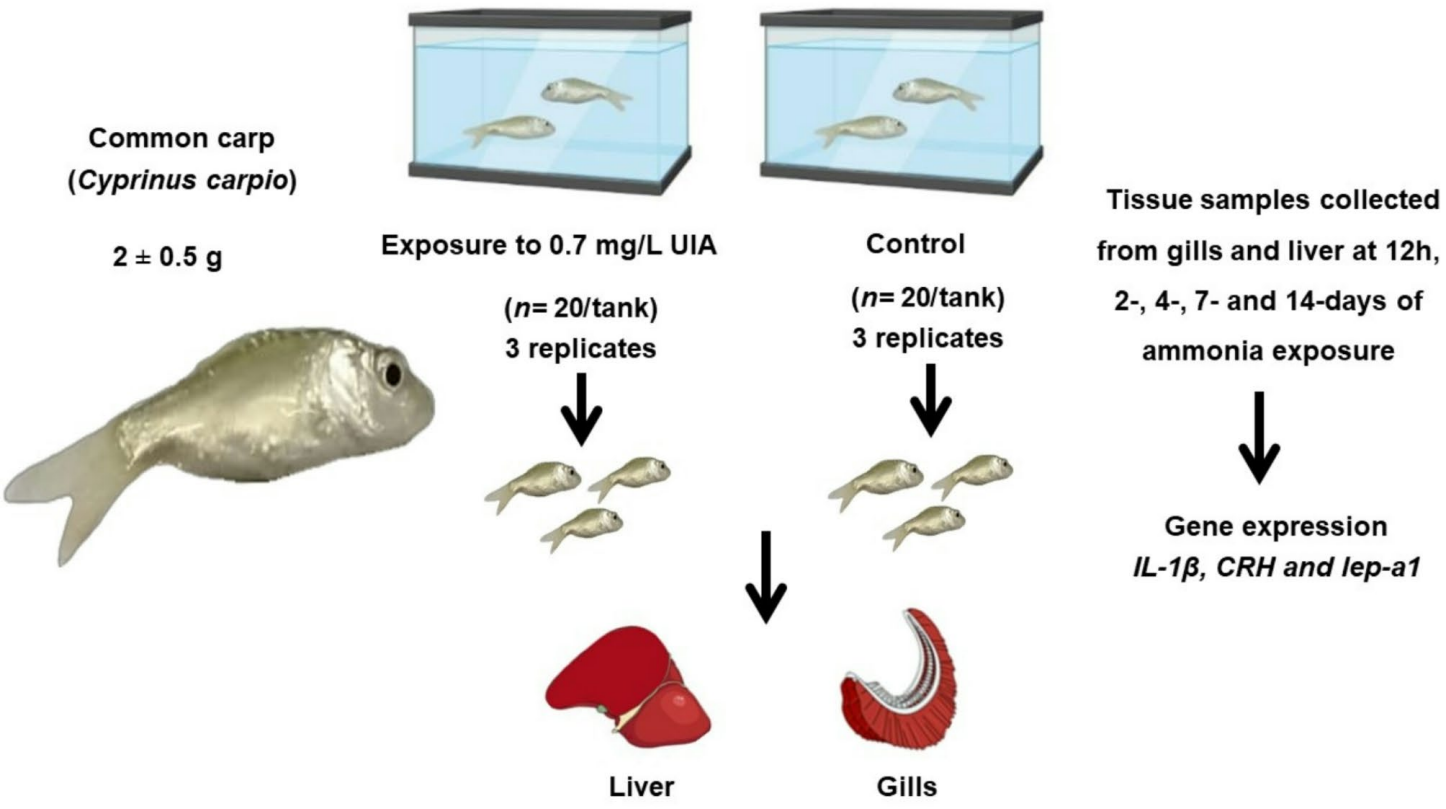
Experimental design framework for studying ammonia exposure in common carp

Fish were euthanized using eugenol (clove oil) at a concentration of 110.1 mg/L, which induced euthanizing within 10 min [43]. Liver and gill samples were collected from three selected fish in each group at 12 h, 2 days, 4 days, 7 days, and 14 days of exposure (Fig. 8).

### RNA extraction and cDNA synthesis

Total RNA was extracted from liver and gill samples using the RNeasy^®^ Mini Kit (Qiagen, Germany). RNA concentration and purity were assessed with a NanoDropTM Lite Spectrophotometer (Thermo Scientific, USA), and samples with a purity of 2.1 ± 0.2 were used for first-strand cDNA synthesis using the RevertAid First Strand cDNA Synthesis Kit (Thermo Scientific, USA). Synthesized cDNA was stored at -20 °C for later use.

### Quantitative real-time PCR (RT-qPCR)

The RT-qPCR was performed using the HERAPlus SYBR^®^ Green QPCR Kit (Willowfort, England) and the CFX96TM RT-PCR Detection System (Bio-Rad, USA). Target genes (*IL-1β*,* CRH*, and *Lep-a1*) and housekeeping genes (*EF1α*, and *β-actin*) were amplified with primers detailed in Table 1. According to the manufacturer’s guidelines, 2 µL of cDNA template, 1 µL of each target gene primer, and 10 µL of 2× SYBR^®^ Green were used for the reaction. The total volume of the reaction was completed to 20 µL by adding sterile PCR-grade nuclease free water. The genes underwent a thermal cycling profile that included an initial denaturation phase at 95 °C for 3 min, denaturation for 40 cycles at 95 °C for 30 s, combined annealing and extension at 60 °C for 1 min, and a final extension at 95 °C for 10 s. During the extension phase, fluorescent data was collected. All reactions contained no template control. The default melting curve analysis was conducted at the end of the amplification process to confirmed amplification specificity. Gene expression fold changes were calculated using the delta-delta Ct method (2^−ΔΔCt^) [44].

**Table 1:** Primer sequences

Gene	Primer (5′-3′)	Annealing temperature	GenBank accession number	Amplicon size (bp)	References
*IL-1β*	F: AAGGAGGCCAGTGGCTCTGT R: CCTGAAGAAGAGGAGGCTGTCA	60 °C	AB010701	69	[45]
*CRH*	F: CATCCGGCTCGGTAACAGAA R: CCAACAGACGCTGCGTTAACT	60 °C	AJ317955	116	[46]
*Lep-a1*	F: CATATTGATTTGTCCACCCTTCTG R: CCATTAGCTGGCTCCTTGGAT	60 °C	AJ868357	83	[46]
*β-actin*	F: GATTCGCTGGAGATGATGCT R: GATGGGGTACTTCAGGGTCA	60 °C	M24113	261	[47]
*EF1α*	F: GGAGCCCAGCACAAACATG R: TTACCCTCCTTGCGCTCAAT	60 °C	AF485331	60	[48]

### Statistical analysis

Data were analyzed using two-way ANOVA with Tukey’s test (*P* < 0.05) in GraphPad Prism 9.5.1. Correlations between gene expressions were evaluated with Past 4.03 software. Results are presented as mean ± standard error of the mean (SEM).

## Conclusion

This study provides valuable insights into the molecular mechanisms underlying the physiological response of common carp to UIA toxicity. The results demonstrate the coordinated upregulation of the inflammatory, stress, and metabolic genes (*IL-1β*,* CRH*, and *Lep-a1*) in both the liver and gills, highlighting the complex and dynamic mechanisms involved in the fish’s adaptive strategies to manage ammonia toxicity. The liver exhibited a more sustained and integrated physiological response, while the gills showed an acute, transient reaction. The gradual recovery of appetite and cessation of mortalities during the second week may be attributed to the development of adaptive mechanisms, such as the upregulation of the urea cycle or glutamine synthesis. The significant positive correlations between the expression of the three genes in both tissues underscores the intricate interconnectedness of the physiological pathways governing the fish’s response to ammonia exposure. These findings contribute to a better understanding of the complex molecular-level impacts of ammonia toxicity on fish and provide valuable insights for improved aquaculture management and environmental conservation.

## Data Availability

No datasets were generated or analyzed during the current study.
